# The Importance of Visual Feedback Design in BCIs; from Embodiment to Motor Imagery Learning

**DOI:** 10.1371/journal.pone.0161945

**Published:** 2016-09-06

**Authors:** Maryam Alimardani, Shuichi Nishio, Hiroshi Ishiguro

**Affiliations:** 1 Department of General Systems Studies, Graduate School of Arts and Sciences, University of Tokyo, Tokyo, Japan; 2 Advanced Telecommunications Research Institute International (ATR), Kyoto, Japan; 3 Department of Systems Innovation, Graduate School of Engineering Science, Osaka University, Osaka, Japan; National University of Defense Technology College of Mechatronic Engineering and Automation, CHINA

## Abstract

Brain computer interfaces (BCIs) have been developed and implemented in many areas as a new communication channel between the human brain and external devices. Despite their rapid growth and broad popularity, the inaccurate performance and cost of user-training are yet the main issues that prevent their application out of the research and clinical environment. We previously introduced a BCI system for the control of a very humanlike android that could raise a sense of embodiment and agency in the operators only by imagining a movement (motor imagery) and watching the robot perform it. Also using the same setup, we further discovered that the positive bias of subjects’ performance both increased their sensation of embodiment and improved their motor imagery skills in a short period. In this work, we studied the shared mechanism between the experience of embodiment and motor imagery. We compared the trend of motor imagery learning when two groups of subjects BCI-operated different looking robots, a very humanlike android’s hands and a pair of metallic gripper. Although our experiments did not show a significant change of learning between the two groups immediately during one session, the android group revealed better motor imagery skills in the follow up session when both groups repeated the task using the non-humanlike gripper. This result shows that motor imagery skills learnt during the BCI-operation of humanlike hands are more robust to time and visual feedback changes. We discuss the role of embodiment and mirror neuron system in such outcome and propose the application of androids for efficient BCI training.

## Introduction

Brain computer interfaces (BCIs) are the new communication devices that translate brain signals into meaningful commands for control of external machines [1–3]. Among different types of BCIs, electroencephalography (EEG)-based BCIs are most commonly used due to their noninvasiveness, high temporal resolution, portability and reasonable cost. There are three main brain activity patterns that are used to design an EEG-based BCI; event-related potentials (ERPs) such as P300 [4–6], steady state visual evoked potentials (SSVEP) [7–9] and event-related desynchornization/synchronization (ERD/ERS) [10, 11]. In recent years, motor related BCIs in which users imagine a movement (motor imagery) and the system detects the corresponding spatial distributions of EEG oscillations (ERD/ERS) have become very popular [12–14]. The rapid improvement of motor imagery BCIs has made them an efficient platform for new developments in such areas as medicine, robotics and entertainment. Today, the BCI technology enables users move prostheses [15–17], navigate through virtual reality [18–20] and control telepresence robotic settings only by thought [21–25]. These advances are indebted to the much effort of BCI researchers who have produced powerful algorithm and high-accuracy classifiers that are responsible for decoding EEG signals. However, with the emerging potentials for using BCIs in various fields and promotion of their real-world application outside of laboratories, there is also a rising need to understand the human side of the interface and identify the opportunities and constraints for this interaction paradigm [26].

There are two major issues that challenge BCI researchers in regard to the human users. The first is user training; the operation of a BCI is not intuitive and users need to learn how to voluntarily control their neural activities [6, 27, 28]. Especially in case of motor imagery based BCIs, a rather long training period is required until the users gain skill in the imagery task and achieve optimal performance. Previous studies have sought various kinds of cognitive tasks and feedback techniques to propose an optimal training protocol [11, 29–31]. In a search for suitable mental task, Neuper et al. showed that motor imagery patterns were only recognizable when the participants used a strategy of kinesthetic motor imagery (first-person process) compared to when they formed a visual image of another’s action (third-person process) [32]. They also evaluated the effect of visual feedback in shape of a realistic moving hand versus an abstract moving bar, however they couldn’t find any significant difference between the two feedback designs [33]. In another study, the comparison of feedback modalities, such as haptic feedback vs. visual feedback, revealed no difference of improvement during BCI control [34]. Only, when both modalities were present and the sensorimotor feedback loop was closed, did the modulation of activities in motor cortex increase [35]. Probably among all the approaches that were taken for training optimization, those that challenged the bias of feedback accuracy were most effective. Although there are some inconsistencies between the results, several groups have shown that positive and negative bias of feedback can influence the performance level of users based on their initial skill levels [36–39].

The second challenging topic for the BCI researchers is in respect to the impact that brain-steered interfaces reflect on the user’s experience of self. In a motor BCI, where imagination of bodily movements (motor imagery) results into the motions of objects other than the user’s real body, the representations of one’s own body may change in a way that the feeling of body ownership and agency is reoriented from the real body toward the controlled device, producing the so-called sense of embodiment [21, 24, 40–42]. Understanding the mechanism of embodiment is particularly important because of the positive impacts it can extend on the BCI control and practicality. For instance, in the case of amputees with a neuro-prosthetic limb, the long-term and efficient usage of the limb depends on how well the patients accept the limb as an integrated part of their own body rather than a tool attached to them [43, 44]. Similarly in the case of BCI-control for a moving avatar or robot, the arousal of embodiment for the user is assumed to promote his involvement in the motor imagery task and enhance his skills in the navigation and operation. So far, studies with virtual reality setup [40, 41] and humanoid robots [21, 23] have investigated the conditions and cognitive process under which the BCI control of another body can elicit a sense of embodiment and ownership in the operators. Recently, Evans and Blanke took a step further and found shared neural underpinnings between motor imagery and illusory body ownership [45]. However, none of the past works has explicitly evaluated the impact of embodiment on the performance of BCI users.

A quick review of the existing literature demonstrates that although research seeks solution to the above issues, user training and embodiment, these studies handle each of them separately and the combination of both is never discussed in any of them. Despite the current trends of merging BCIs with virtual reality and robotic setups [46] that has facilitated the human-interface interaction, surprisingly to our knowledge, there is no work that has explicitly assessed the impact of embodiment on motor imagery learning during BCI control.

In this paper, our approach aims at bridging these two problems, by introducing a potential relationship between the sense of embodiment during BCI-control and the improvement of user’s BCI performance. We previously proposed a BCI operational system for a very humanlike android robot (called Geminoid), which through its realistic appearance could induce a strong sense of body ownership transfer (BOT) in the operators [21]. We also demonstrated that by designing a suitable feedback paradigm and manipulating the subjects’ perception of BCI performance, operators could engage with the motor imagery task easier and optimize their regulation of brain activities for a better BCI control [36, 37]. However to what extent the BOT illusion and the match between the visual feedback from robot with one’s experience of body control contributed to the optimized learning of motor imagery remained undiscussed. In this work, we further compare the effect of feedback design on motor imagery learning between the android and a non-humanlike robot (a pair of metallic gripper), to seek the exact impact of robot’s appearance and discuss how the sense of embodiment and body ownership illusion can play an important role in the improvement of motor imagery skills.

## Methods

### Ethics statement

Experiments in this study were conducted in full accordance with the ethical guidelines of Advanced Telecommunications Research Institute International (ATR) and the Ethics Committee of the named institute specifically approved this study. (Approval number: 13-506-3).

### Participants

Thirty-eight healthy, right-handed subjects (17 male and 21 female, age *M* = 23.8, *SD* = 8.2) participated in this experiment. None of them had participated in our previous experiments and they were all beginners with BCI system. Participants received explanation prior to the experiment and signed a written consent form.

### BCI-Teleoperation

The teleoperational interface developed in this work consisted of a BCI system and two different robots.

**Fig 1:**
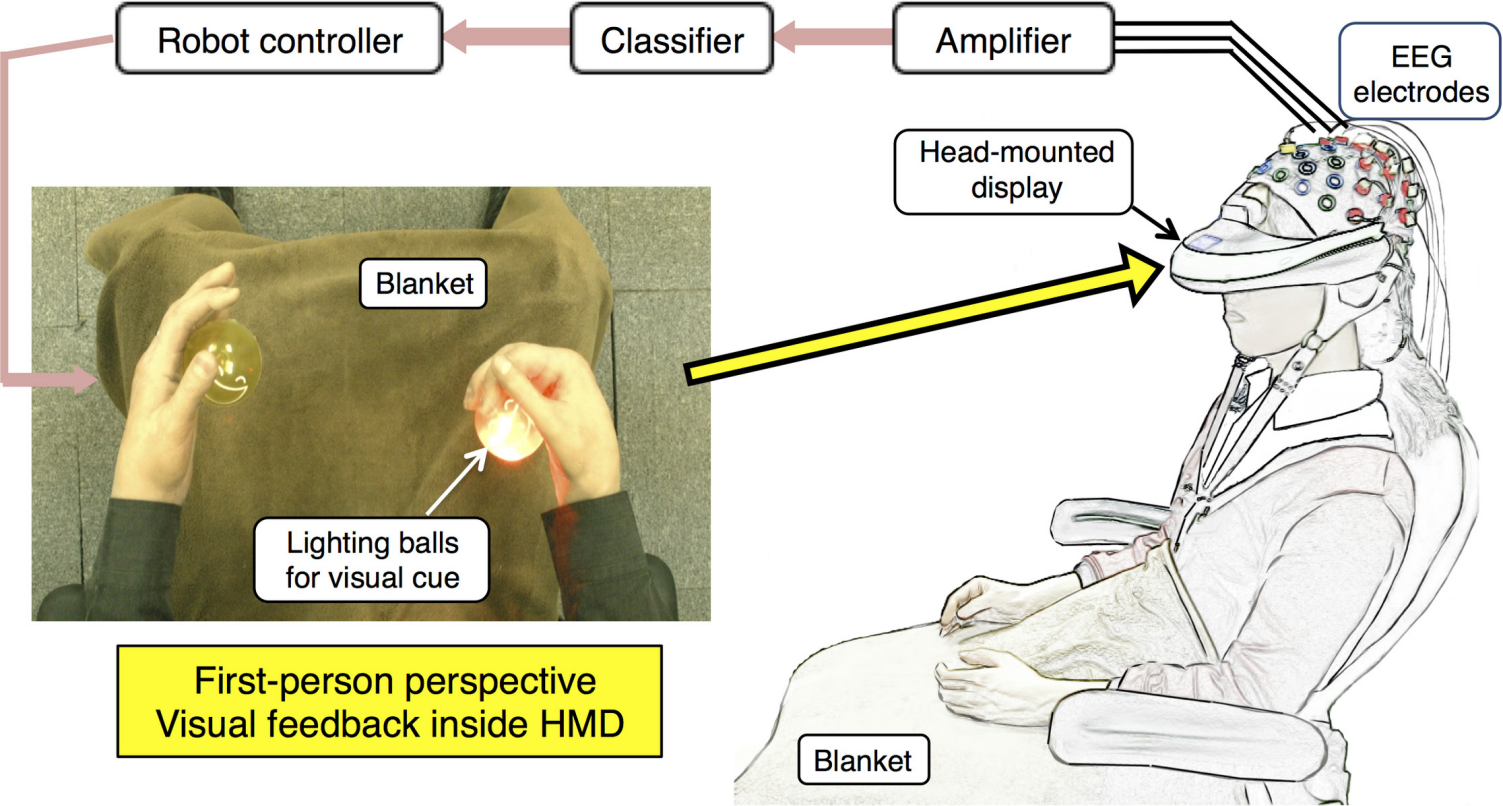
Experiment setup. 27 EEG electrodes recorded brain activities during a motor imagery task. Subjects watched first-person images of a robot’s hands through a head mounted display. A lighting ball in front of the robot’s hands gave motor imagery cue and subjects held images of a grasp for their own corresponding hand. Classifier detected two classes of results (right or left) and sent a motion command to the robot’s hand.

The BCI system recorded 27 EEG channels over the primary sensori-motor cortex, referenced to the right ear and grounded to the forehead. Participants performed a motor imagery task for their own right and left hand while they watched through a head-mounted display (Vuzix iWear VR920) a first-person perspective image of the robot’s hands (Fig 1). Two balls were placed inside the robot’s hands. Each time the right or left ball was lighted, participants held an image of grasp for the corresponding hand. Cerebral activities were recorded by g.USBamp biosignal amplifiers (Guger Technologies) and the BCI classifier translated the motor imagery patterns into motion commands for the robot’s hands. The classification of recorded signals was conducted under Simulink/MATLAB (Mathworks) for offline and online parameter extraction. This process included bandpass filtering between 0.5 and 30 Hz, sampling at 128 Hz, cutting off artifacts by notch filter at 60 Hz, and adopting the Common Spatial Pattern (CSP) algorithm for discrimination of Event Related Desynchronization (ERD) and Event Related Synchronization (ERS) patterns associated with the motor imagery task [47]. Results were classified with weight vectors that weighed each electrode based on its importance for the discrimination task and suppressed the noise in individual channel by using the correlations between neighboring electrodes. During each right or left imagery movement, the decomposition of the associated EEG led to a new time series, which was optimal for discrimination of two populations. The patterns were designed such that the signal from the EEG filtering with CSP had maximum variance for the left trials and minimum variance for the right trials and vice versa. In this way, the difference between the left and right populations was maximized and the only information contained in these patters was where the EEG variance fluctuated the most during the comparisons between the two conditions. Finally when the discrimination between left and right imaginations was made, the classifier outputted a linear array signal in the range of [–1, 1], where -1 was commanded as a full left grasp and 1 was commanded as a full right grasp [21].

**Fig 2:**
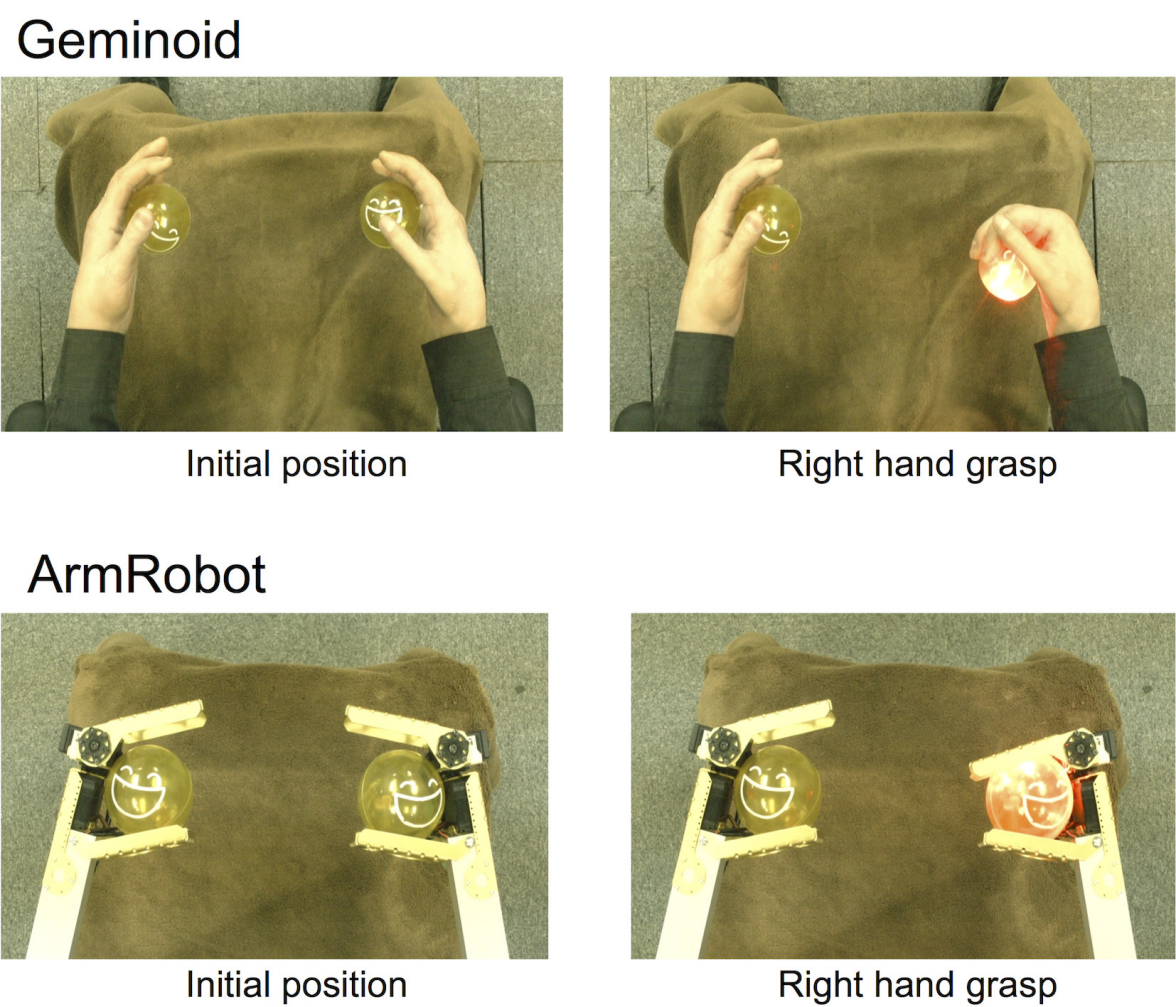
Geminoid vs ArmRobot. Two different robots, a pair of very humanlike android hands versus a pair of robotic arms were used for BCI-teleoperation to evaluate the effect of humanlikeness and body ownership illusion on motor imagery learning.

The robots used in this experiment, Geminoid and ArmRobot, are shown in Fig 2. During the visual feedback of motion, Geminoid used four fingers to grasp the lighted ball and ArmRobot used only the upper prong to clutch the ball. Identical blankets were laid on both the subjects’ legs and the robots’ legs to match the body background view. In this experiment, we used an edited video of the robot’s motions, however when at the end of the experiment, participants were questioned if they noticed anything unusual in the images or that the video they watched was not a real time video but a previously recorded and edited video, the response from all participants was negative.

### Procedure

After receiving instructions about the motor imagery task, participants sat in a chair and experimenter placed EEG electrodes. All participants performed one session of non-feedback calibration (for the setup of subject-specific classifier) and one session of training with a normal computer screen. Following to this, they wore head mounted display and performed another two sessions of BCI-teleoperation with the robots (Session 3 and Session 4).

All sessions included 40 trials, 20 trials per class left/right in a randomized order. Each trial lasted 7.5 seconds and included an acoustic warning at second 2 for the onset of imagery task. From second 3 to 4.25, a visual cue in form of an arrow on the screen during the training session or a lighting ball inside the robot’s hand during the teleoperational sessions was presented based on which the subjects had to hold an image of grasp for their right or left hand. From second 4.25 to 7, the feedback was presented to the subject. In the training session, the feedback was in form of a horizontal bar on the screen extending to right or left. In the teleoperational sessions, robot’s hands made a grasp based on the online result of the BCI classifier.

After completing the first and second non-operational sessions, participants were separated into two groups of nineteen members and each group used different robot for BCI-operation in Session 3 (Fig 2). In Session 4, again all of the participants used the same robot, ArmRobot, for BCI-operation. Therefore, the experimental conditions for each group can be summarized as below:

1. Geminoid group: Participants initially operated Geminoid’s hands in Session 3 and proceeded to operation of the ArmRobot in Session 4.
2. ArmRobot group: Participants BCI-operated only the robotic arms in both Session 3 and Session 4.

**Fig 3:**
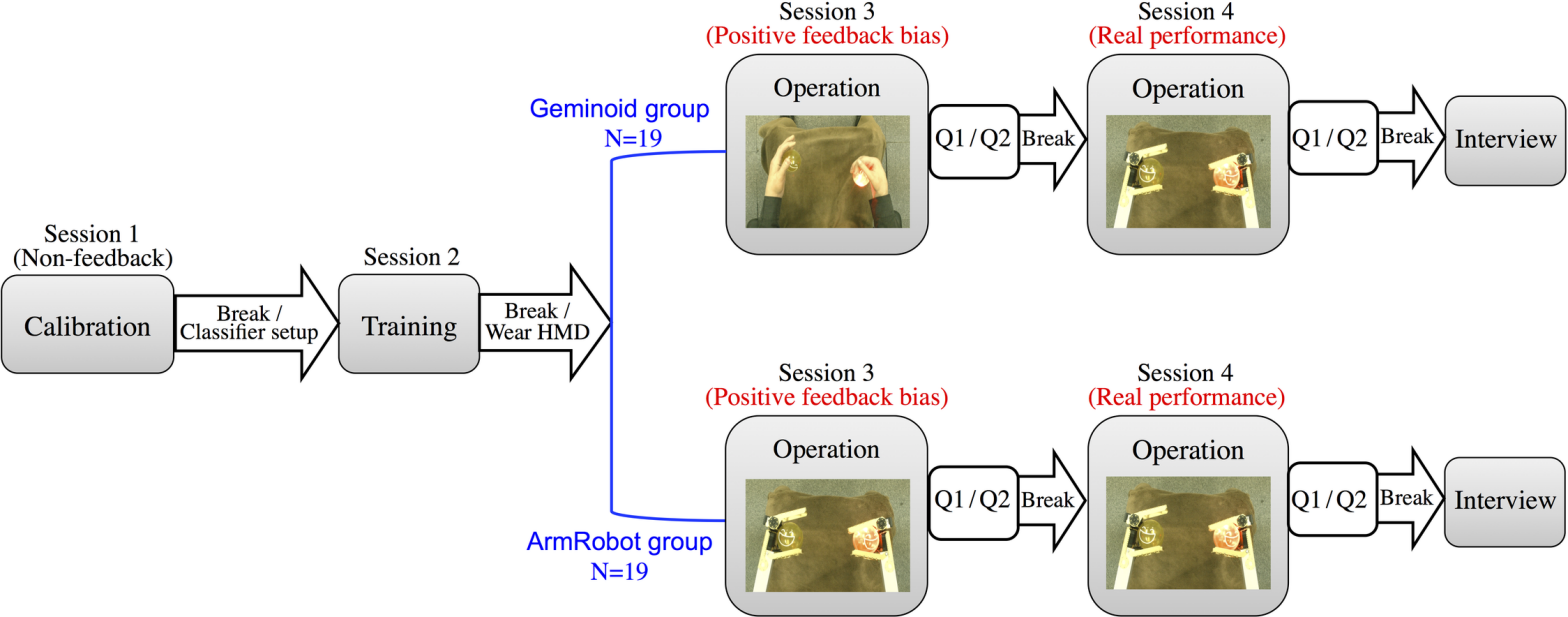
Experimental procedure. Subjects performed one session of non-feedback calibration (for the setup of subject-specific classifier) and one session of training with a normal computer screen. Then, they wore head-mounted display and prepared for BCI-teleoperation of the robots. Subjects were divided into two groups: (i) Geminoid group was first trained with a humanlike robot and then operated a pair of metallic gripper. (ii) ArmRobot group only used the metallic grippers for operation in both Sessions 3 and 4. All sessions included 40 trials. Subjects answered a questionnaire at the end of each teleopreational session.

Similar to our previous work regarding the effect of biased feedback on the motor imagery leaning [36, 37], participants of both groups received a positive bias of feedback (90% correct) in the first half of Session 3. The goal here was to compare the impact of the same feedback design between two different looking robots. In Session 4 however, feedback accuracy was not conditioned and subjects received their real performance that was outputted from the classifier. The aim of designing Session 4 was to measure the robustness of the motor imagery skills learnt by each robot in Session 3 and estimate if optimized motor imagery patters sustain for BCI-operation of other robots, once they are learnt. An illustration of the experiment procedure is depicted on Fig 3.

At the end of each BCI-operational session participants responded orally to the BOT questionnaire (refer to Evaluation). Also participants commented about their performance in each session during an interview following the experiment termination. Particularly the Geminoid group was asked to compare the feasibility of motor imagery task between the two sessions with different robots.

### Evaluation

Since we examined the effect of body ownership illusion on the motor imagery learning during BCI-teleoperation, it was important to measure the subjective experience of BOT illusion for each robot separately. Therefore evaluation was made by two methods: 1) questionnaire to measure the self-assessed BCI performance and level of BOT illusion, 2) offline analysis of brain activities for estimation of the motor imagery improvement.

#### 1) Questionnaire

The questionnaire we asked from the subjects included two primary questions on the self-estimated performance and ownership illusion:

Q1) Could you operate the robot’s hands according to your intentions?
Q2) Did you feel as if the robot’s hands were your own hands?

Subjects scored the above questions on a 7-point Likert scale, 1 denoting “couldn’t operate / didn’t feel at all” and 7 denoting “could operate very well / felt very strongly”.

#### 2) Motor imagery learning and robustness

We evaluated motor imagery learning based on how well the subject could generate brain patterns discriminant for the two classes of right and left hand grasps during the operation. Since our BCI system used a previously setup classifier for the recognition task and did not use a learning algorithm, the online performance of the subjects could not serve as a reliable measure. Because the subjects may have consciously or unconsciously modified the generation of their brain activity patterns and in that case the classifier would fail to detect these changes. Therefore, we used the original brain signals and conducted an offline analysis of brain activities to extract the feature distribution of right and left motor imagery trials in each session. We then used Fisher’s discriminant criterion measures in a linear discriminant analysis to observe the distribution of the two classes’ feature vectors in a 4-dimential space (see details in Ref. [36, 37]). Fisher’s parameter J is defined as

$$J=\frac{{\left|{\tilde{\mu }}_{R}-{\tilde{\mu }}_{L}\right|}^{2}}{{\tilde{s}}_{R}^{2}+{\tilde{s}}_{L}^{2}}$$ (1)

where the quantity ${\left|{\tilde{\mu }}_{R}-{\tilde{\mu }}_{L}\right|}^{2}$ is the distance between the two classes’ means and the quantity ${\tilde{s}}_{R}^{2}+{\tilde{s}}_{L}^{2}$ indicates the within-class scatter. When performing motor imagery, a larger *J* corresponds to a closer dispersion of feature vectors per each class and a further distance between two class means, which represents a more discriminant distribution of feature vectors within the feature space and therefore the better execution of motor imagery task by subject.

The trend of motor imagery learning in Session 3 was defined as the ratio between the class separability in the second 20 test trials (*J*_2_) and the first 20 biased trials (*J*_1_). Therefore Δ*J* = *J*_2_/*J*_1_ was selected as a measure of motor imagery (MI) learning due to the elicit of BOT illusion.

$$\Delta J_{MI\;learning}=\frac{J_{2}}{J_{1}}$$ (2)

In addition, *J* parameter was compared between Session 3 and Session 4 to estimate the robustness of the online motor imagery skills in Session 4, once they are acquired due to the feedback bias and BOT illusion in Session 3. The class separability was calculated for the 40 trials in Session 4 (*J*_4_) and for the 40 trials in Session 3 (*J*_3_). The ratio between these two parameters

$$\Delta J_{MI\;robustness}=\frac{J_{4}}{J_{3}}$$ (3)

was selected as the measure of motor imagery durability and robustness when the illusion was shattered and subjects proceeded to the BCI-teleoperation of another robot in a different paradigm.

## Results

Subjects’ answers to the questionnaire and the results of motor imagery learning measure Δ*J* is provided in the following two sections.

### 1) Questionnaire

**Fig 4:**
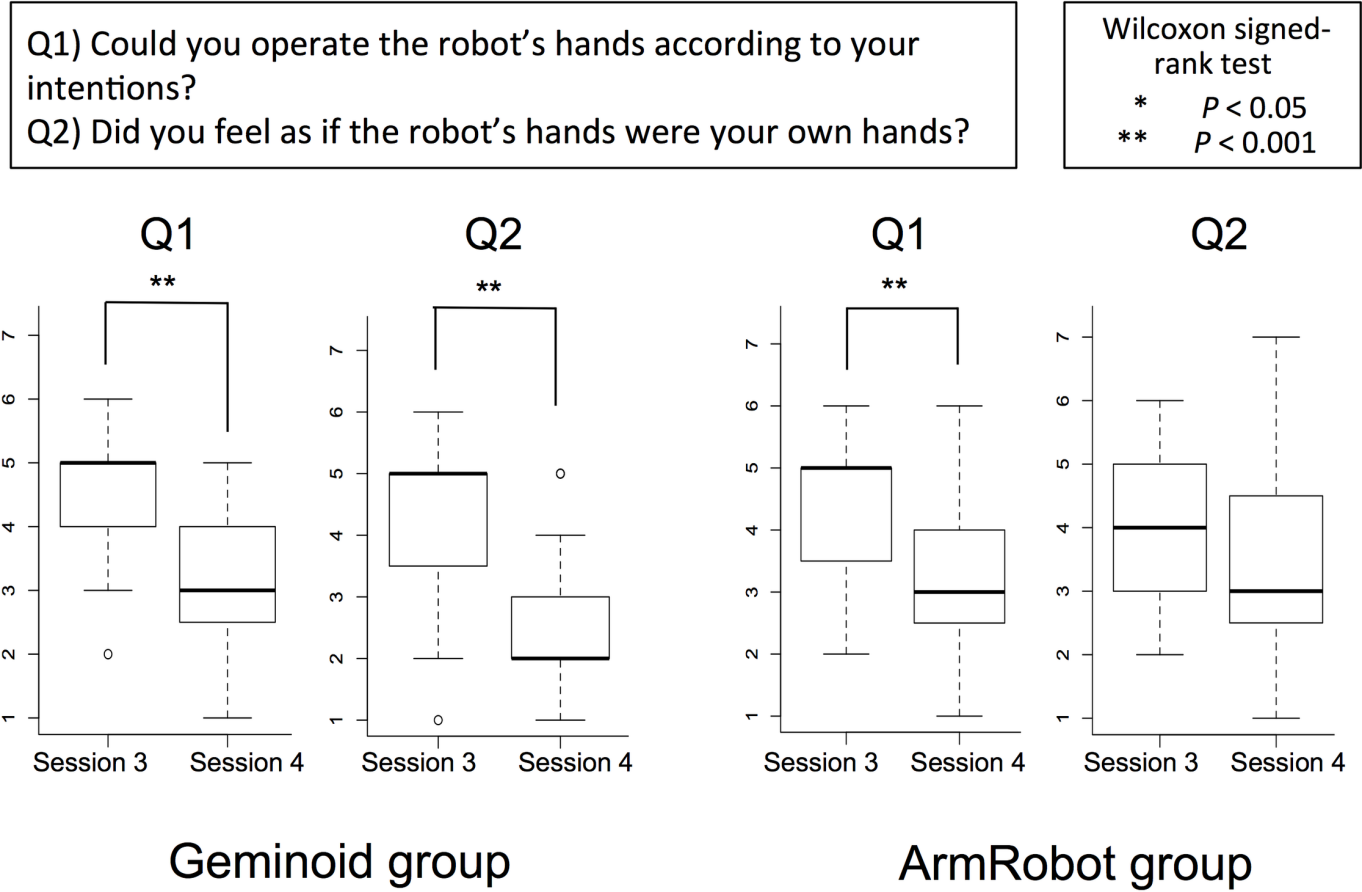
Questionnaire results. Median values and interquartile ranges for Q1 (self-evaluated performance) and Q2 (body ownership illusion) at the end of Session 3 and Session 4.

Participants’ responses to the questionnaire for both Gemionid and ArmRobot group were averaged and compared between the two sessions, Session 3 and Session 4. The mean value, standard deviation, and p-value for each group are depicted on Fig 4.

For both Q1 and Q2, the responses from both Geminoid and ArmRobot group showed a higher mean value in Session 3 compared to Session 4. Since the Likert scores are of ordinal nature, we used a non-parametric statistical test to analyze our results (S1 Table). For the Geminoid group, a Wilcoxon signed-rank test on scores of Q1, which asked about the operators’ self-evaluated performance during the session, showed a significant difference between Session 3 (M = 4.58, SD = 1.17) and Session 4 (M = 3.05, SD = 0.97); [Session 3 > Session 4, *Z* = 3.45, *p* < 0.001]. The same test on Q1 scores from ArmRobot group revealed a significantly higher score in Session 3 (M = 4.47, SD = 1.22) than Session 4 (M = 3.21, SD = 1.23); (Session 3 > Session 4, *Z* = 3.48, *p* < 0.001).

On the other hand, Wilcoxon signed-rank test on responses from Geminoid group to Q2, which directly asked the intensity of BOT over the operated hands, displayed significantly higher score in Session 3 (M = 4.36, SD = 1.38) than Session 4 (M = 2.53, SD = 1.17) [Session 3 > Session 4, *Z* = 3.74, *p* < 0.001], whereas the responses from ArmRobot group to Q2 in Session 3 (M = 4.0, SD = 1.11) and Session 4 (M = 3.53, SD = 1.61) did not reveal a significant difference [*Z* = 1.46, *p* = 0.18], although it was comparatively higher in Session 3, perhaps due to the effect of positive bias during the first half of Session 3.

### 2) Motor imagery learning and robustness

**Fig 5:**
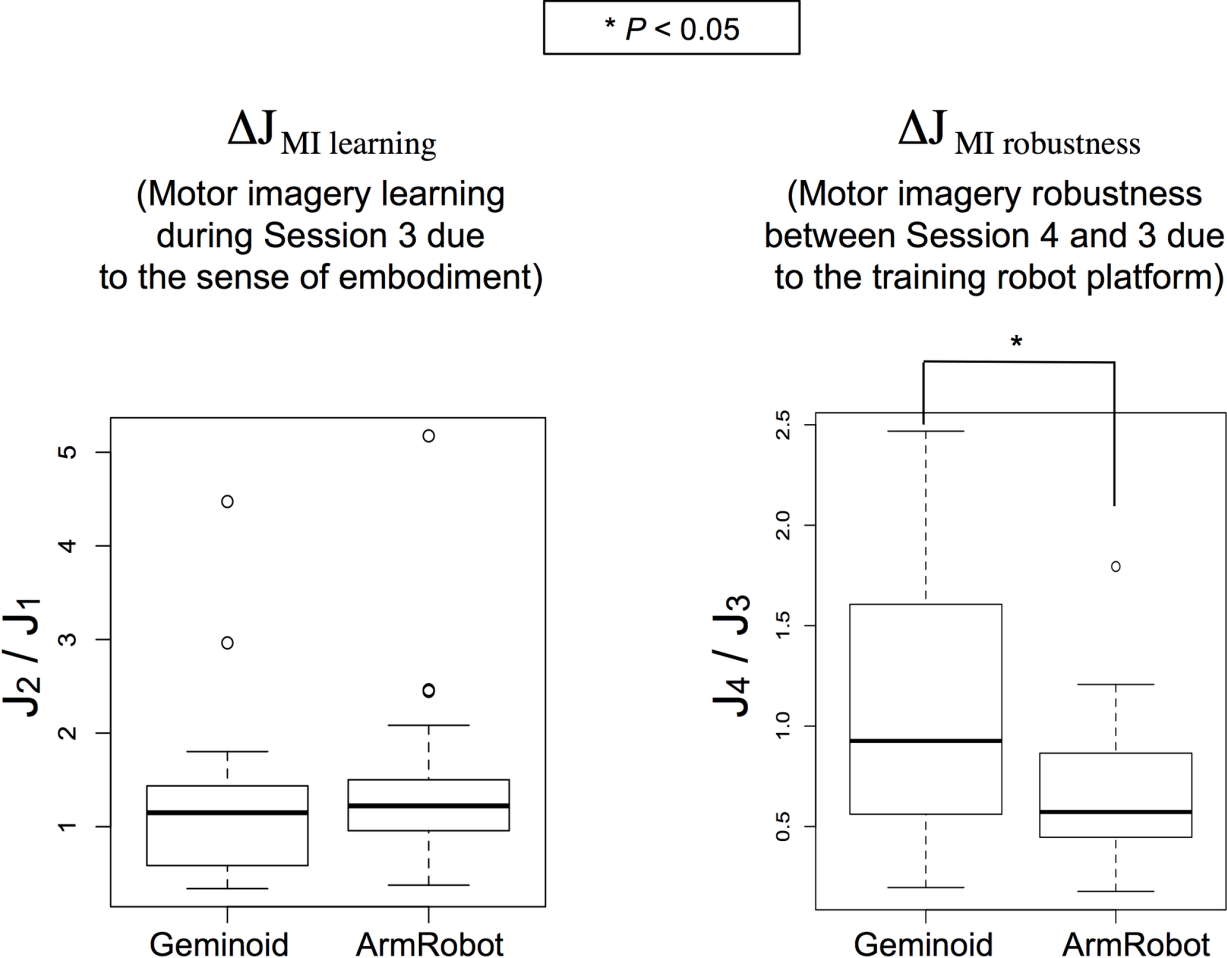
Results for EEG offline analysis. Δ*J* (a measure of right/left class separation in the feature space during the motor imagery task) was calculated between two halves of Session 3 to evaluate the trend of motor imagery learning due to the BOT illusion. Also, Δ*J* was compared between Session 3 and Session 4 to estimate the robustness of motor imagery skills learnt through each robot. Median values and interquartile ranges are depicted on each graph.

For each group of participants, the results of Δ*J*_MI learning_ and Δ*J*_MI robustness_ are demonstrated in Fig 5.

We used the Shapiro-Wilk test to evaluate whether the data (S2 Table) were normally distributed. The results from this test for the *J*_2_/*J*_1_ data (*W* = 0.94, *p* = 0.25), and *J*_4_/*J*_3_ data (*W* = 0.98, *p* = 0.96) did not reject the null hypothesis that the data are normally distributed. Therefore, assuming normal distribution, two-sample t-test was used to compare the results between the two groups of participants.

The comparison of ΔJ_MI learning_ during Session 3 (ratio *J*_2_/*J*_1_) did not show a significant difference between the two Geminoid group (M = 1.31, SD = 0.97) and ArmRobot group (M = 1.48, SD = 1.05) [*t*(36) = -0.11, p = 0.91]. Whereas, the comparison of Δ*J*_MI robustness_ between Session 3 and Session 4 (ratio *J*_4_/*J*_3_) showed a significantly higher mean value for the Geminoid group (M = 1.08, SD = 0.7) than the ArmRobot group (M = 0.68, SD = 0.42); [Geminoid > ArmRobot, *t*(36) = 2.15, p < 0.05], however the mean value for *J*_4_/*J*_3_ ratio was approximately 1 for Geminoid group and it dropped to less than 1 for the ArmRobot group, indicating a decreasing effect on *J* parameter.

## Discussion

The present study was particularly conducted to investigate the impact of visual feedback and sense of embodiment on the motor imagery skills acquired during BCI-control. Two groups of novice subjects BCI-operated different looking robots; one group operated a very humanlike android robot (Geminoid) in the first session and then a pair of metallic gripper (ArmRobot) in the second session. The other group practiced operation with only ArmRobot in both sessions. The results did not show a significant improvement in motor imagery learning immediately in the first operational session, however they revealed a significant robustness of motor imagery skills in the following session for only the Geminoid group, who reported a significantly stronger sense of embodiment for the android robot compared to the metallic gripper.

A comparison between the motor imagery performance calculated by our J parameter in Session 3 and Session 4 showed that for subjects who first BCI-operated android hands in one session and then proceeded to the operation with robotic arms, the Δ*J*_MI robustness_ remained significantly higher than the other group who practiced motor imagery only with the robotic arms in both sessions. This result suggests that the motor imagery skills learnt during a biased training session with a humanlike robot could sustain for the following BCI session with a different robot, even though the feeling of embodiment for the new robot (Q2 scores in Fig 4) dropped significantly. However, the same training paradigm with a less humanlike robot did not show such robustness and a mean value of less than 1 for the ratio *J*_4_/*J*_3_ indicates a decrease in motor imagery performance for the ArmRobot group.

The significant decline of performance in Session 4 may initially appear unusual, however it can be explained as following based on the conditioning of the operational sessions; In Session 3, subjects received positively biased visual feedback in the first half and then continued to the unbiased (real performance) trials. In Session 4 on the other hand, subjects performed all trials under unbiased condition, which means they received visual feedback of their real performance as it was outputted from the BCI classifier. Since in an unbiased BCI-control, the unsuccessful imagery attempt of subjects for the target hand was presented as an opposite grasp for the robot’s other hand, the interference between observation and self-imagined movements could intensely disturb the motor imagery task and drop the level of confidence and concentration in subjects. This effect could be also confirmed for both Geminoid and ArmRobot group as their scores to Q1 dropped significantly from Session 3 to Session 4 (Fig 4). Hence, a suppression of *J* parameter was obviously expected in both groups due to the change of performance accuracy in visual feedback. Nevertheless, Geminoid group could resist the decline of *J* and hold on their skills for operation even in an unbiased session.

The reason for this effect could be discussed based on the mechanism under which motor imagery is practiced and the changes it reflects on the motor circuitry and cortical excitability during the process of learning. Motor imagery is defined as a dynamic state during which representations of a certain motor act are internally rehearsed in the working memory without motor action [48–50]. Several studies have provided evidence that motor imagery can produce changes, as powerful as those produced by motor execution, at both behavioral and neural level [13, 51–55]. Ehrsson et al. showed that during motor imagery, the content of mental image is reflected on the cortical activation and imagery of certain body parts such as toe, finger, and tongue could result into the activation of different areas in the primary motor cortex [56]. Another work has shown that perspective and posture of imagery task can also generate a difference, that is, the first-person perspective imagery leads to a stronger activation in the motor-related structures [57]. In addition to these evidences for the effect of imagery content on the cortical activity, the content of visual feedback during the performance of motor imagery has also been shown to interact with motor processes, thereby modulating the excitability in the motor cortex. In an assessment of how visual feedback about posture affects the level of activation during hand motor imagery, Mercier et al. showed that looking at a hand posture compatible with the imagined movement resulted in an increase of cortico-spinal excitability, facilitation of motor representations in memory and regulation of central motor imagery processes [58]. Similarly, the works on mirror therapy for patients who suffer from hemiplegia or phantom pain has shown that watching the unaffected hand in a mirror assisted the motor imagery of the affected hand and resulted in a significant increase of the motor evoked potentials compared to the motor imagery without mirror [59].

Altogether, the above literature suggest that motor imagery includes a visuo-spatial representation regulated by working memory and similar to motor action it can produce changes in brain plasticity [60]. During the performance of motor imagery, the content of both self-induced image and the visual feedback can affect the activation level of motor cortex and induction of neural pathways. In this study, the difference revealed by the two groups’ performance in the second part of the experiment therefore suggests a difference in the level of cortical excitations each group experienced. The Geminoid group who watched a humanlike hand with a more detailed appearance and compatible action may have experienced a higher excitation of motor processes that induced efficient connectivity and significant changes in neural plasticity, which thus resulted facilitation in learning. Therefore, one can assume that even in the absence of the humanlike robot, the Geminoid group recalled more vivid and explicit representations of the imagery task and performed better on the BCI-operation.

A rather unexpected result was obtained in the first part of this experiment, where the comparison of Δ*J*_MI learning_ did not reveal a significant improvement in motor imagery learning between the two groups. A main reason that may have yielded this outcome is probably our failure in the estimation of humanlikeness for the ArmRobot and underestimating its potential in inducing the ownership illusion. We initially developed ArmRobot as a non-humanlike robot to be compared versus Geminoid as an android with an extremely humanlike appearance. However, the experiment results contradicted our assumption. The obtained Q2 scores (Fig 4) for the ArmRobot group (who did not experience operational sessions with Geminoid) showed surprisingly high effect in Session 3 in terms of embodiment (almost same as Geminoid group in Session 3) and this effect did not change significantly in Session 4. Whereas, the Q2 score obtained from the Geminoid group (who initially observed Geminoid and then proceeded to the operation of the ArmRobot) had a significant decrease from Session 3 to Session 4. Thus, we can recognize that the perception of humanlikeness and body ownership illusion was relative in this case, based on the subjects’ previous experience of robots. In other words, those who already operated the Geminoid, found the ArmRobot significantly less humanlike and reported weaker embodiment, whereas the second group who did not have the chance to make a comparison between the robots’ appearances could experience a strong embodiment for the moving ‘metallic hands’.

It has been previously shown that such ownership illusions as rubber hand illusion [61] that are raised by multisensory integration can only be induced for humanlike body parts and not the non-corporeal objects [62]. However, in the case of motion and voluntary action, body ownership is not the only component that contributes to the mechanism of self-recognition and embodiment. The sense of agency, which is the feeling of being the generator of the action, can also play a major role in establishing the sense of embodied self. In the present study, beside the anthropomorphic kinematics, the matching of goal and timing of the ArmRobot motions with the subject’s intention raised a high sense of agency that could lead to a strong embodiment in the operators of the ArmRobot.

Another cause for the indifferent results between the two groups could be the impact of action observation and strong firing of mirror neurons in both conditions [63]. The BCI classifier monitors the suppression of sensorimotor brain rhythms in order to recognize the left and right classes. However, in this design there are two components that produce the suppression of brain activities; one is the motor imagery task or mental simulation of hand grasp, the other is viewing the action of robot and activation of the mirror neurons system [64, 65]. There is evidence that mirror neurons are more sensitive to some motions than others, for instance observation of a hand precision grasp can cause greater suppression of mu rhythm than observation of a flat-hand extension [66]. Moreover, the presence of an object (here the ball inside robot’s hand), indicating the goal of action, increases the mu rhythm suppression as compared to meaningless motions [67]. Therefore, the overlapping activity of motor imagery and action observation during experiment may have generated ERD patterns in sensorimotor area that are not necessarily distinguishable.

Another reason for the lack of significant difference in the learning progress of the two groups could be the inadequate training. Learning to modulate brain activities to control a BCI is a difficult task that requires attention, motivation and above all practice [6, 27]. In the present study, we recruited novice BCI users who had their first encounter with a BCI system and performed only one training session (Session 2) before the test sessions. For these subjects, the unfamiliarity of the motor imagery task and the tension or stress caused by the experimental environment may have been the psychological factors that influenced the subjects’ learning potentials, causing the reduced impact of the embodiment parameter on the learning progress. Larger number of training sessions, performed in regular intervals, would customize the inexperienced users with a BCI experiment and therefore optimize their capacity for learning [33].

Summing up, the use of a humanlike robot for the BCI control of hand grasps could generate a strong sense of embodiment and induce long-term and robust motor imagery skills. These findings are supported by the outcome of (1) questionnaire, where the users self-rated their performance and feeling of body ownership, and (2) analysis of EEG activities and motor patterns where the segregation of EEG features for each motor imagery class was measured. Our results can further provide evidence for a close linkage between the visual and the motor processes, especially under conditions in which no movement occurs. Visual inputs are recognized as the more effective source for motor learning compared to proprioceptive feedback [68], particularly in the early stages of learning [69]. Therefore, it is speculated that during motor imagery where the sense of proprioception is absent, the role of visual feedback in facilitation or inhibition of motor representation becomes further dominant [58]. Hence, taking into account the importance of visual feedback design is critical in the development of efficient training protocols for motor imagery based BCIs.

## Conclusions

In this work, we combined an EEG-based BCI with two types of different looking robots to investigate the shared mechanism of embodiment and motor imagery. We hypothesized that the learning of motor imagery task for the BCI-control can be enhanced if the visual feedback is aligned with our every day experience of human body. We recruited two groups of subjects who BCI-controlled a pair of very humanlike android hands versus a pair of metallic gripper. Our aim was to purposefully create different levels of body ownership illusion and therefore examine the effect of stronger embodiment on the operator’s motor imagery learning.

Although our results did not show a significant improvement of motor imagery skills between two groups during the first biased session, in a follow-up session when all participants operated the robotic gripper, the group who initially practiced motor imagery task with the android’s hands showed significantly higher BCI controlling skills than the group who only experienced the operation of robotic gripper. This shows that motor imagery skills learnt during the BCI-operation of humanlike hands are robust to time and visual feedback changes. Future experiment should re-challenge these findings with a completely non-humanlike robot to investigate the effect of BOT illusion on motor imagery learning in an online session.

Altogether, by suggesting a positive interaction between the inducement of embodiment and improvement of subjects’ motor imagery skills, our findings reveal the promising nature of BCI-teleoperation for an efficient interaction through android robots and furthermore the application of such system in the future BCI training paradigms.

## Supporting Information

S1 Table Questionnaire responses. Raw data collected from the subjects to Q1 and Q2.
(PDF)

S2 Table J values in each session. (PDF)
