# Supplementary material for: The Importance of Visual Feedback Design in BCIs; from Embodiment to Motor Imagery Learning
Source: PLoS One. 2016 Sep 6;11(9):e0161945. doi: 10.1371/journal.pone.0161945 (PMC5012560; doi:10.1371/journal.pone.0161945)
Supplement: S1 Table — Raw data collected from the subjects to Q1 and Q2. (PDF) [file pone.0161945.s001.pdf]

S1 Table. Questionnaire responses.

Geminoid Group			
Session3		Session4	
Q1	Q2	Q1	Q2
4	6	2	1
3	4	3	2
5	3	3	1
6	5	4	3
5	6	4	5
5	6	3	2
3	5	2	3
5	3	2	2
5	5	3	2
3	5	4	4
5	4	3	3
5	4	1	2
6	5	4	3
2	2	3	2
4	3	3	2
5	1	2	1
4	5	3	3
6	5	5	5
6	5	4	2

ArmRobot Group			
Session3		Session4	
Q1	Q2	Q1	Q2
5	3	3	3
6	3	4	2
3	4	2	2
5	5	3	3
6	5	4	5
5	4	2	2
3	4	2	3
5	3	4	4
6	5	6	6
3	5	4	5
5	5	4	4
4	4	3	4
6	5	4	7
3	4	1	1
2	2	1	1
4	6	4	5
5	4	3	3
5	3	3	4
4	2	4	3

Geminoid group		
Session	Q1	Q2
Session3	4	6
Session3	3	4
Session3	5	3
Session3	6	5
Session3	5	6
Session3	5	6
Session3	3	5
Session3	5	3
Session3	5	5
Session3	3	5
Session3	5	4
Session3	5	4
Session3	6	5
Session3	2	2
Session3	4	3
Session3	5	1
Session3	4	5
Session3	6	5
Session3	6	5
Session4	2	1
Session4	3	2
Session4	3	1
Session4	4	3
Session4	4	5
Session4	3	2
Session4	2	3
Session4	2	2
Session4	3	2
Session4	4	4
Session4	3	3

ArmRobot group		
Session	Q1	Q2
Session3	5	3
Session3	6	3
Session3	3	4
Session3	5	5
Session3	6	5
Session3	5	4
Session3	3	4
Session3	5	3
Session3	6	5
Session3	3	5
Session3	5	5
Session3	4	4
Session3	6	5
Session3	3	4
Session3	2	2
Session3	4	6
Session3	5	4
Session3	5	3
Session3	4	2
Session4	3	3
Session4	4	2
Session4	2	2
Session4	3	3
Session4	4	5
Session4	2	2
Session4	2	3
Session4	4	4
Session4	6	6
Session4	4	5
Session4	4	4

Session4	1	2
Session4	4	3
Session4	3	2
Session4	3	2
Session4	2	1
Session4	3	3
Session4	5	5
Session4	4	2

Session4	3	4
Session4	4	7
Session4	1	1
Session4	1	1
Session4	4	5
Session4	3	3
Session4	3	4
Session4	4	3
