# Supplementary material for: The Importance of Visual Feedback Design in BCIs; from Embodiment to Motor Imagery Learning
Source: PLoS One. 2016 Sep 6;11(9):e0161945. doi: 10.1371/journal.pone.0161945 (PMC5012560; doi:10.1371/journal.pone.0161945)
Supplement: S2 Table — (PDF) [file pone.0161945.s002.pdf]

S2 Table. J values in each session.

Geminoid group						
	J1 (20 trials) Session3-1st half	J2 (20 trials) Session3-2nd half	J2/J1 2nd half vs 1st half	J4 (40 trials) Session4	J3 (40 trials) Session3	J4/J3 Session4 vs Session3
Sub1	0.0869	0.1148	1.321058688	0.0137	0.0369	0.371273713
Sub3	0.0552	0.0941	1.704710145	0.0216	0.0254	0.850393701
Sub5	0.0587	0.08	1.36286201	0.0167	0.0247	0.67611336
Sub7	0.1069	0.131	1.225444341	0.0094	0.0496	0.189516129
Sub9	0.0567	0.0609	1.074074074	0.0118	0.0197	0.598984772
Sub11	0.0283	0.0232	0.819787986	0.0225	0.007	3.214285714
Sub13	0.0258	0.0208	0.80620155	0.011	0.006	1.833333333
Sub15	0.0797	0.0558	0.700125471	0.0173	0.0228	0.75877193
Sub17	0.073	0.1647	2.256164384	0.0022	0.0231	0.095238095
Sub19	0.0319	0.0246	0.771159875	0.016	0.0093	1.720430108
Sub21	0.0568	0.0126	0.221830986	0.0109	0.0059	1.847457627
Sub23	0.0467	0.0344	0.736616702	0.0073	0.0153	0.477124183
Sub25	0.0431	0.0567	1.315545244	0.0068	0.0204	0.333333333
Sub27	0.0734	0.1678	2.286103542	0.0074	0.0319	0.231974922
Sub29	0.0428	0.0282	0.658878505	0.0087	0.0099	0.878787879
Sub31	0.0281	0.0787	2.800711744	0.0227	0.0111	2.045045045
Sub33	0.0235	0.0809	3.442553191	0.0228	0.0229	0.995633188
Sub35	0.0474	0.053	1.11814346	0.0278	0.0116	2.396551724
Sub37	0.0336	0.0557	1.657738095	0.0144	0.0136	1.058823529

ArmRobot group						
	J1 (20 trials) Session3-1st half	J2 (20 trials) Session3-2nd half	J2/J1 2nd half vs 1st half	J4 (40 trials) Session4	J3 (40 trials) Session3	J4/J3 Session4 vs Session3
Sub2	0.0439	0.0328	0.74715262	0.0184	0.0375	0.490666667
Sub4	0.1042	0.1113	1.068138196	0.0074	0.0322	0.229813665
Sub6	0.0769	0.0544	0.707412224	0.0358	0.0406	0.881773399
Sub8	0.0684	0.0865	1.264619883	0.0065	0.0218	0.298165138
Sub10	0.0202	0.0231	1.143564356	0.0083	0.005	1.66
Sub12	0.0289	0.0695	2.404844291	0.0053	0.0099	0.535353535
Sub14	0.0714	0.0489	0.68487395	0.0035	0.0173	0.202312139
Sub16	0.0294	0.0224	0.761904762	0.0066	0.0055	1.2
Sub18	0.0084	0.0337	4.011904762	0.0058	0.0031	1.870967742
Sub20	0.0852	0.0998	1.171361502	0.013	0.0349	0.372492837
Sub22	0.0436	0.0965	2.213302752	0.0165	0.0213	0.774647887
Sub24	0.1089	0.1045	0.95959596	0.0124	0.0348	0.356321839
Sub26	0.026	0.0291	1.119230769	0.0116	0.0265	0.437735849
Sub28	0.0324	0.059	1.820987654	0.015	0.0191	0.785340314
Sub30	0.0724	0.1035	1.429558011	0.0103	0.0225	0.457777778
Sub32	0.0286	0.0487	1.702797203	0.0039	0.037	0.105405405
Sub34	0.086	0.1364	1.586046512	0.0069	0.0157	0.439490446
Sub36	0.0972	0.0993	1.021604938	0.0345	0.023	1.5
Sub38	0.0572	0.0588	1.027972028	0.0063	0.0137	0.459854015
